# Translational Inhibition of Slug by Pdcd4 Contributes to Invasion Inhibition in Colorectal Cancer Cells

**DOI:** 10.1002/cam4.71145

**Published:** 2025-08-11

**Authors:** Qing Wang, Wei He, Shilong Han, Maoquan Li, Yanlei Wang, Yekaterina Zaytseva, Li Chen, Jiang Zhu, Hsin‐Sheng Yang

**Affiliations:** ^1^ Department of Toxicology and Cancer Biology, College of Medicine University of Kentucky Lexington Kentucky USA; ^2^ Department of General Surgery Qilu Hospital of Shandong University Jinan Shandong Province China; ^3^ Shanghai Tenth People's Hospital of Tongji University Shanghai China; ^4^ Markey Cancer Center, College of Medicine University of Kentucky Lexington Kentucky USA

**Keywords:** E‐cadherin, eIF4A, invasion, Pdcd4, Slug colorectal cancer

## Abstract

**Background:**

Colorectal cancer (CRC) metastasis remains a major cause of mortality, driven by epithelial‐to‐mesenchymal transition (EMT) and invasion. Programmed cell death 4 (Pdcd4), a tumor suppressor, is known to inhibit translation via interaction with eukaryotic initiation factor 4A (eIF4A). Previous studies have established that Pdcd4 suppresses stress‐activated protein kinase 1‐interacting protein 1 (Sin1) translation through the mTORC2‐Akt axis, thereby downregulating Snail expression and EMT in CRC cells. However, whether Pdcd4 directly regulates Slug, another critical EMT transcription factor, remains unexplored.

**Method:**

*PDCD4* shRNA and *SLUG* siRNA were used to knock down Pdcd4 and Slug in colorectal cancer cells, respectively. The sucrose gradient fractionation was performed to determine *SLUG* translation. A luciferase reporter assay was used to determine the role of the *SLUG* 5′ untranslated region (5'UTR) on Pdcd4 inhibition. The effect of Slug on promoting invasion was determined by Matrigel invasion assays.

**Result:**

Knockdown of Pdcd4 in colorectal cancer cells increased Slug protein levels without altering *SLUG* mRNA abundance. Sucrose gradient fractionation revealed that Pdcd4 knockdown elevated the proportion of *SLUG* mRNA in polysome fractions, demonstrating Pdcd4‐mediated suppression of *SLUG* translation. To validate the mechanism, the *SLUG* 5′UTR was cloned and fused to a luciferase reporter and named *SLUG*‐5′UTR‐Luc. Pdcd4 knockdown markedly enhanced SLUG‐5′UTR‐Luc activity; whereas, ectopic Pdcd4 expression suppressed it, indicating that the *SLUG* 5′UTR is critical for Pdcd4‐mediated translational repression. Treatment with the eIF4A inhibitor silvestrol substantially reduced Slug protein levels and SLUG‐5′UTR‐Luc activity. In addition, Pdcd4 overexpression decreased Slug protein abundance and restored E‐cadherin expression. Notably, Slug knockdown in Pdcd4‐deficient cells rescued E‐cadherin expression and abrogated the invasive phenotype. These findings suggest that up‐regulation of Slug translation by Pdcd4 knockdown contributes to enhanced invasion.

**Conclusion:**

Pdcd4 suppresses colorectal cancer invasion by translationally downregulating Slug expression.

**Abbreviations**
eIF4A: eukaryotic translation initiation factor 4A
EMT: epithelial‐to‐mesenchymal transition
FRPs: free RNAs and proteins
mTORC2: rapamycin complex 2
p70S6K1: p70 ribosomal protein S6 kinase 1
Pdcd4: Programmed cell death 4
*TCGA*‐*COAD*
: The Cancer Genome Atlas Colon Adenocarcinoma
TIC: translation initiation complex
u‐PAR: urokinase‐type plasminogen activator receptor
XIAP: X‐linked inhibitor of apoptosis

## 1 Introduction

Colorectal cancer represents one of the most commonly diagnosed and devastating cancers. Despite clinical advances in the treatment of early‐stage colorectal cancer, distant metastasis remains the main cause of the low five‐year survival rate (13%–18%), whereas patients with localized disease have a significantly higher survival rate (~90%) [1]. Metastasis is a complex process in that cancer cells spread from a primary tumor to a secondary site within the human body. To initiate this metastatic dissemination, cancer cells have to detach from a primary tumor and invade the surrounding tissue by destructing cell–cell junctions, remodeling cell‐matrix adhesion sites, and secreting proteinases to pass through the extracellular matrix [2]. This process often requires that cancer cells transfer from epithelial phenotype to mesenchymal phenotype (epithelial‐to‐mesenchymal transition, EMT) to gain cell motility [3]. E‐cadherin, a calcium‐dependent cell–cell adhesion glycoprotein, frequently loses function during the EMT process [3]. E‐cadherin expression is regulated by various mechanisms, including promoter methylation and histone deacetylation. In addition, the zinc‐finger transcription repressors, the Snail family (Snail and Slug), have been stated to repress E‐cadherin expression, leading to the gain of invasive capability in various cancer cells [4, 5]. Snail and Slug suppress E‐cadherin transcription by binding to the enhancer box element in the proximal E‐cadherin promoter [6], thereby dismantling cell–cell junctions and promoting mesenchymal phenotypes. In addition to regulating cell motility, Snail and Slug also play important roles in maintaining other cellular functions. For example, Snail has been reported to confer resistance to apoptosis by downregulating pro‐apoptotic factors and upregulating survival signals [7]. In contrast, Slug uniquely enhances cancer stem cell properties by activating stemness markers such as SOX9 and OCT4 [8] and promotes tolerance to genomic instability through interactions with DNA repair pathways [9]. These divergent roles highlight their complementary contributions to EMT progression. A recent study also suggested that Slug may modify histone by recruiting histone deacetylase 6 to suppress E‐cadherin expression [10]. Ectopic expression of Slug in colorectal cancer cells induces EMT and enhances invasive capability [5]. Conversely, knockdown of Slug by *SLUG* siRNA restores E‐cadherin expression and suppresses invasion [11]. In colorectal cancer patients, the level of Slug expression is positively associated with cancer stage progression and metastasis [12, 13], indicating that Slug promotes colorectal cancer progression. Importantly, patients with positive expression of Slug showed the worst prognosis in colorectal cancer [12], gastric cancer [14], and breast cancer [15].

Programmed cell death 4 (Pdcd4), a tumor suppressor, is ubiquitously expressed in all of the tissues and is frequently down‐regulated in various types of cancers including colorectal cancer. Pdcd4 has been demonstrated to inhibit cell proliferation [16] and overcome drug resistance [17]. In addition, Pdcd4 also plays an important role in regulating tumor invasion and metastasis. For example, knockdown of Pdcd4 expression using *PDCD4* shRNA results in fibroblast‐like phenotype transformation due to increased expression of mesenchymal markers (β‐catenin and N‐cadherin) and decreased expression of epithelial markers (E‐cadherin and α‐catenin), and thereby enhances migration and invasive capability [18, 19]. Conversely, over‐expression of *PDCD4* cDNA suppresses intravasation and invasion [20, 21]. In agreement with these in vitro studies, Pdcd4 knockdown promotes metastasis in colorectal cancer HT29 and GEO cells when these cells were injected into the cecal wall of nude mice [19]. Moreover, down‐regulation of E‐cadherin by Pdcd4 knockdown is partially attributed to the elevation of Snail expression via stress‐activated‐protein kinase 1 (Sin1)‐mTORC2‐Akt axis (details see the recent review [22]). However, the effect of Pdcd4 on the expression of Slug, the other member of the Snail family, is unknown.

Another biological function of Pdcd4 is inhibiting protein translation, which has been demonstrated to attenuate the translation of Sin1 [23], p70 ribosomal protein S6 kinase 1 (p70S6K1) [24], p53 [25], X‐linked inhibitor of apoptosis (XIAP) [26], and c‐Myb [27]. Using yeast two‐hybrid, Pdcd4 was initially identified to physically interact with translation initiation factor 4A (eIF4A). Later, the Pdcd4‐eIF4A interaction is further confirmed by crystallography analysis showing that Pdcd4 uses two MA‐3 domains to bind with eIF4A [28]. By binding with eIF4A, Pdcd4 inhibits the helicase activity of eIF4A and thereby suppresses protein translation [29]. The eIF4A is an ATP‐dependent RNA helicase that unwinds the structured mRNA, allowing the translation initiation complex to scan along the mRNA and search for the translation start codon [30]. Therefore, the inhibition of eIF4A helicase activity by Pdcd4 is expected to attenuate protein translation, especially the translation of mRNAs with stable secondary structure at the 5′untranslated region (5′UTR) [31, 32].

In this study, we aimed to understand the impact of Slug on Pdcd4 knockdown‐induced colorectal cancer cell invasion and elucidate the mechanism by which Pdcd4 regulates Slug expression.

## 2 Materials and Methods

### 2.1 Chemicals and Reagents

The eIF4A inhibitor, silvestrol, was acquired from MedChemExpress (Monmouth Junction, NJ) and dissolved in dimethyl sulfoxide (Sigma) at 1 mM and stored at −20°C. The final concentration used for cell treatment is indicated in the figure legends.

### 2.2 Cell Lines and Culture Conditions

Colorectal cancer HT29 and carcinoma RKO cells were purchased from American Type Culture Collection. The primary colorectal cancer Pt130 cells (passage 5) were a gift from Dr. Tainyan Guo at University of Kentucky [33]. Cells were grown in McCoy's medium containing 10% FBS, 2 mM L‐glutamine, and 100 U/mL penicillin–streptomycin. All cells were cultured at 37°C with 5% CO_2_ in a humidified incubator.

### 2.3 Western Blot Analysis

Cell lysate (30–100 μg) was separated on an SDS‐PAGE and transferred to a nitrocellulose membrane as described previously [34]. Subsequently, the membrane was incubated with primary antibodies overnight at 4°C, followed by incubation with horseradish peroxidase‐linked secondary antibodies (1:2000 dilution, Cell signaling). The target protein was visualized by Immobilon Western Chemiluminescent HRP substrates (Millipore) and detected by Azur c600 Gel Imaging System (Azure Biosystems). The antibodies against Slug (1:1000 dilution, #9585), HA (1:2000 dilution, #3724), and E‐cadherin (1:1000 dilution, #3195) were purchased from Cell Signaling. The Snail antibody (1:1000 dilution, #13099–1‐AP) was obtained from Proteintech. The GAPDH antibody was obtained from Santa Cruz Biotechnology (1:5000 dilution, sc‐47,724). The Pdcd4 antibody was generated as described previously [18].

### 2.4 Sucrose Gradient Fractionation

Sucrose Gradient Fractionation was performed as previously described [23]. Briefly, cells were grown to 70%–80% confluence. The cells were incubated with 10 μg/mL cycloheximide for 10 min before incubation with lysis buffer [24]. After centrifugation, the supernatant was loaded on 10%–45% sucrose and centrifuged at 36,000 rpm (160,000 × g at γ_av_) for 2 h at 4°C in an SW41Ti rotor. Each fraction was collected by Piston Gradient Fractionator (Biocomp) and monitored at 254 nm by Econo UV monitor (Bio‐Rad). Data were acquired by Gradient Profiler software (Biocomp).

### 2.5 RNA Extraction and Real Time‐PCR

For total RNA, cells were lysed with Trizol (Invitrogen) and total RNA was isolated by Direct‐zol RNA Miniprep Kit (Zymo Research). For fractionation of RNAs, liquid samples were mixed with Trizol evenly at a ratio of 1:3, and RNAs were isolated using Direct‐zol RNA Miniprep Kit and subsequently precipitated with 7.5 M LiCl overnight.

Real‐time PCR was performed as described previously [35]. Briefly, total RNA (1 μg) or fraction RNA (500 ng) was used to synthesize the first‐strand cDNA with the Superscript First‐Strand Kit (Invitrogen). The mRNA levels of Slug, Snail, ATF4, or GAPDH were quantified by real‐time PCR in a LightCycler 480 (Roche Applied Science). All primers used for amplifying were pre‐designed and purchased from Integrated DNA Technologies (Snail: Hs.PT.58.2984401, Slug: Hs.PT.58.1772559, Pdcd4: Hs.PT.58.22664674, ATF4: Hs.PT.56a.744435.g, and GAPDH: HsPT.39a.22214836). The relative levels of target mRNA (i.e., *PDCD4*, *SLUG, SNAIL*, or *ATF4*) were calculated by the 2^−ΔΔCt^ method. The Ct values of triplicates of target mRNA and *GAPDH* mRNA in each sample were used in the formula ΔCt = Ct(target) − Ct(GAPDH). The relative level of *PDCD4*, *SLUG, SNAIL*, or *ATF4* mRNA was determined by comparison of *PDCD4*, *SLUG, SNAIL*, or *ATF4* mRNA in the experimental group to that in the corresponding control group using the formula 2^−[DCt(target)−DCt(control)]^.

### 2.6 5′ RACE (Rapid Amplification of cDNA Ends) and Construction of Plasmids

5′ RACE was performed to clone the *SLUG* 5′ UTR sequence by using SMARTer RACE cDNA Amplification Kit (Clontech) following the manufacturer's instructions. Briefly, after isolating total RNA, 500 ng of total RNA was used to synthesize SMARTer first strand cDNA library, following two rounds of PCR amplification using Platinum Taq DNA Polymerase High Fidelity (Invitrogen). The first round of PCR was performed by a universal primer (5′‐CTAATACGACTCACTATAGGGCAAGCAGTGGTATCAACGCAGAGT‐3′) provided by the kit and the primer (5′‐CCTGAGCTGAGGATCTCTGGTTGTGGTATG‐3′) located at 122 bp of *SLUG* gene translation start site. The second round PCR was performed using the diluted amplified product from the first round PCR as a template and nested universal primer (5′‐AAGCAGTGGTATCAACGCAGAGT‐3′) and the GSP primer provided by the kit. The second round PCR product was then cloned into pcDNA3.3‐TOPO TA Cloning vector (Invitrogen) and verified by DNA sequencing. The *SLUG* 5′ UTR was subsequently cloned into NheI and HindIII sites of pCMV‐Luc plasmid [31] and named SLUG‐5′UTR‐Luc.

### 2.7 Transient‐Transfection and Luciferase Activity Assays

HT29, RKO, or Pt130 cells (5 × 10^4^) were seeded in a 24‐well plate and transfected with 0.2 μg of SLUG‐5′UTR‐Luc, pCMV‐Luc, or E‐cadherin promoter reporter (E6‐Luc) along with 20 ng of pRL‐TK and 0.0–1.5 μg of Pdcd4 expression plasmid (pcDNA3.1‐Pdcd4). Total DNA was normalized to 1.7 μg by adding empty vector (pcDNA3.1). For in vivo translation assays, 16–18 h post‐transfection, cells were serum‐starved (0% FBS) for 24 h, followed by incubation in complete medium with 10% FBS for an additional 24 h. For E‐cadherin promoter activity assays, cells were harvested 48 h after transfection. Cells were lysed in the passive lysis buffer (Promega), and the luciferase activity was measured as previously described [35].

### 2.8 Knockdown of Pdcd4 or Slug

For knockdown of Pdcd4, cells were incubated with lentiviral particles (MOI = 20) containing *PDCD4* shRNA or *LacZ* shRNA for 24 h and subsequently selected by Blasticidin (8 μg/mL) for 14 d. The *PDCD4* shRNA (5′‐CTGGAAGTACCTCATTTTC‐3′) and the *LacZ* shRNA (5′‐GGTAACAGTCTTGGCGGTTTC‐3′) were cloned into the lentivirus expression vector using the BLOCK‐iT Lentiviral RNAi Expression System (Invitrogen) according to the manufacturer's protocol. Lentiviral particles containing *PDCD4* shRNA and *LacZ* shRNA were generated by transfecting 293FT cells with 3 μg of *PDCD4* shRNA or *LacZ* shRNA expression plasmid along with 9 μg of ViraPower Packaging Mix (Invitrogen). The viral particles were collected 48 h post‐transfection. For Slug knockdown, cells (5 × 10^5^) were transfected with 110 pmole of *SLUG* siRNA using INTERFERin siRNA transfection reagent (Polyplus). Forty‐eight to seventy‐two hours post‐transfection, the cells were collected for downstream applications. The pre‐designed *SLUG* siRNA was purchased from Ambion (AM16104) and Integrated DNA Technologies (hs.RiSNAI2.13.1). The negative control siRNA was purchased from Ambion (AM4611).

### 2.9 Matrigel Invasion Assay

Cells were incubated in McCoy's medium without FBS for 24 h. Then, 1 × 10^5^ cells were suspended in McCoy's medium and seeded onto a Matrigel‐coated Transwell chamber (8‐μm pore size; Corning). Epidermal growth factor (20 ng/mL) diluted in McCoy's medium containing 0.5% FBS was added to the lower chamber. The chambers were incubated at 37°C for 24 h. Non‐migrated cells on the upper surface of the membrane were removed using cotton swabs. The membrane was then fixed and stained with 1% (w/v) crystal violet in ethanol. Migrated cells were quantified by counting cells in seven randomly selected fields.

### 2.10 Pdcd4, Snail, and Slug Expression Analysis From Databases

For comparison of Snail and Slug in normal and cancerous tissues, the mRNA level of normal (*n* = 41) and tumor (*n* = 275) normalized transcripts per kilobase million (TPM) mapped reads from The Cancer Genome Atlas Colon Adenocarcinoma (*TCGA*‐*COAD*) were downloaded and analyzed. Wilcoxon rank sum test was used to compare the difference between normal and tumor tissues. For correlation analyses, the mRNA expressions in colorectal cancer (*n* = 521) in the format of upper quartile of fragments per kilobase of transcript per million (FPKM) mapped reads from TCGA‐COAD were downloaded. Spearman's correlation coefficient was used to quantify the correlations between Pdcd4 and Slug as well as Pdcd4 and Snail expressions. The reverse phase protein array (RPPA) level 3 protein expression data (*n* = 327) were downloaded from The Cancer Proteome Atlas (TCPA) website (https://tcpaportal.org/). A detailed description of RPPA level 3 data is available on the website. Spearman's correlation coefficient was used to quantify the correlations between Pdcd4 and Snail as well as Pdcd4 and Slug expressions. The Kaplan–Meier curve and log‐rank test were used to compare overall survival between groups. The biomarker low group is defined as biomarker value ≤ median; the biomarker high group is defined as biomarker value >median.

### 2.11 Statistical Analysis

The levels of *SLUG* mRNA, E‐cadherin promoter activity, and invasive capability were analyzed by a two‐sample student *t*‐test to compare the control group and experimental group. The band intensities of Western blots were analyzed by a one‐sample *t*‐test. Data were expressed as mean ± SD, and *p* < 0.05 was considered significant. The *p* value and the number of replicates are displayed in figure legends.

## 3 Results

### 3.1 Snail Expression Is Inversely Correlated With Pdcd4 Expression in Colorectal Cancer

**FIGURE 1:**
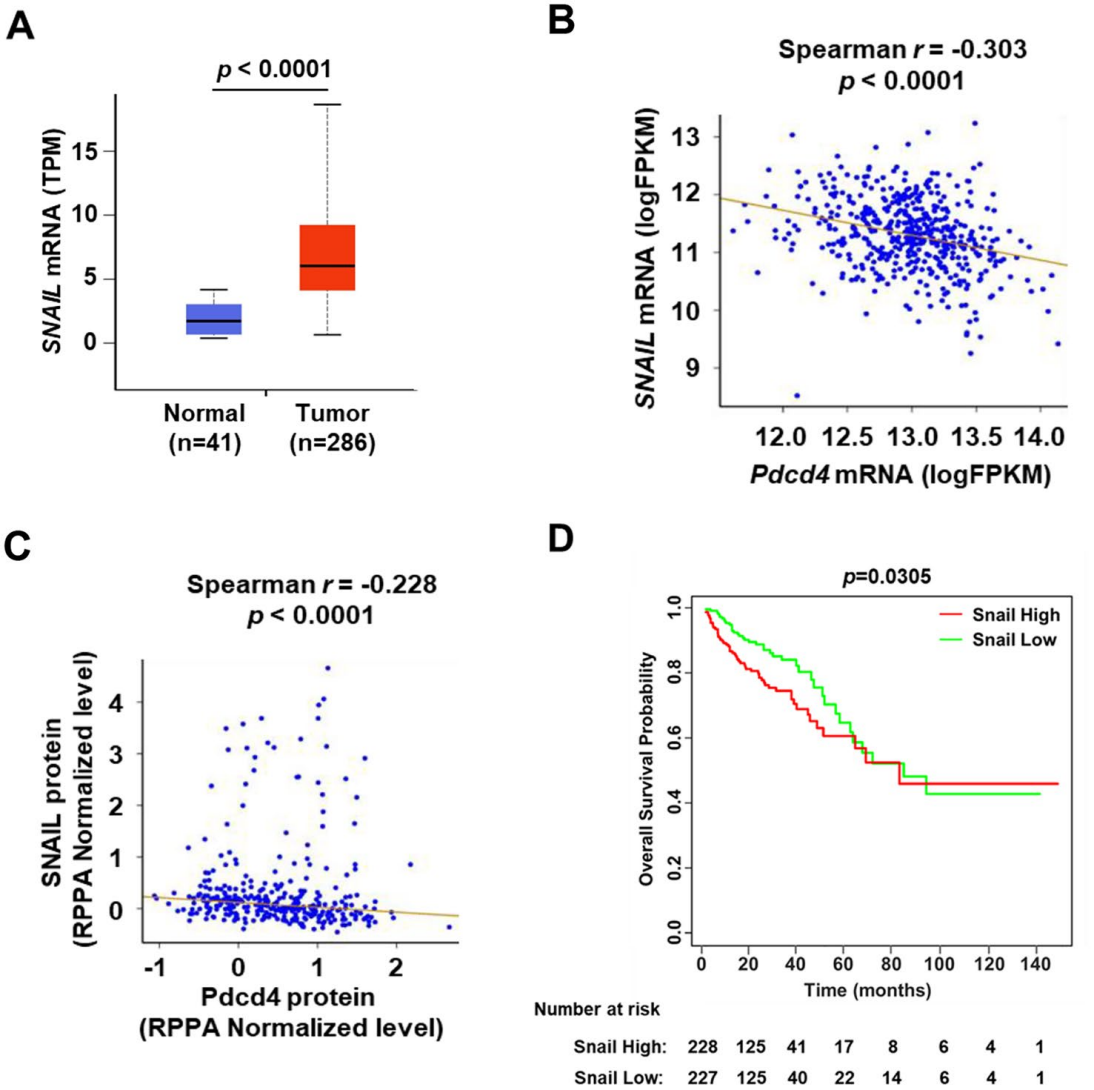
Snail expression in normal and cancer colon tissues. The data of mRNA and protein level were downloaded from TCGA‐COAD and TCPA, respectively. (A) The *SNAIL* mRNA is up‐regulated in colon cancerous tissues. The normalized value of *SNAIL* mRNA was used for statistical analysis by two‐sample *t* test (*p* < 0.0001). (B) The level of *SNAIL* mRNA is inversely correlated with *PDCD4* mRNA. The normalized values of *SNAIL* mRNA and *PDCD4* mRNA were used for statistical analysis by Spearman rank correlation test (r = −0.303; *p* < 0.0001). (C) The protein level of Snail is also inversely correlated with the Pdcd4. The Snail and Pdcd4 protein levels (RPPA Normalized Expression Level) were analyzed by Spearman rank correlation test (r = −0.228; *p* < 0.0001). (D) Kaplan–Meier overall survival curves and number at risk table for patients with low and high *SNAIL* mRNA levels. The Kaplan–Meier curve and log‐rank test were used to compare the overall survival between high and low *SNAIL* mRNA groups (*p* = 0.031). The estimated 1‐year, 2‐year, 3‐year, 5‐year, and 8‐year survival rates for the *SNAIL* mRNA low group are 92.5%, 88.7%, 84.0%, 64.7%, and 42.8%, respectively, compared to 86.2%, 77.7%, 71.9%, 60.6%, and 45.9% for the *SNAIL* mRNA high group.

Snail and Slug are two important transcription factors to regulate EMT [36] and Pdcd4 is a critical inhibitor for EMT in colorectal cancer [18, 19]. To investigate the clinical relevance of Snail, Slug, and Pdcd4, we analyzed their mRNA expression levels using data from the TCGA‐COAD database. The *SNAIL* mRNA was significantly elevated in the colorectal cancerous tissues compared with those in the normal tissues (Figure 1A) and the *SNAIL* mRNA level was significantly negatively correlated with the *PDCD4* mRNA level [Spearman's correlation coefficient = −0.303, 95% confidence interval = (−0.383, −0.210), *p* < 0.0001] (Figure 1B). In agreement with the *SNAIL* mRNA results, the Snail protein was also inversely correlated with the Pdcd4 protein level [Spearman's correlation coefficient = −0.228, 95% confidence interval = (−0.330, −0.113), *p* < 0.0001] (Figure 1C). These findings are in agreement with our previous studies, in which Pdcd4 expression is frequently down‐regulated in colorectal cancer patients [23] and Pdcd4 knockdown up‐regulates Snail expression [23]. In addition, a higher Snail mRNA level has a significant impact on patient overall survival by Kaplan–Meier and log‐rank test (*p* = 0.031, Figure 1D). However, there is no significant difference in overall survival between *PDCD4* mRNA high and low groups (*p* = 0.582, data not shown).

**FIGURE 2:**
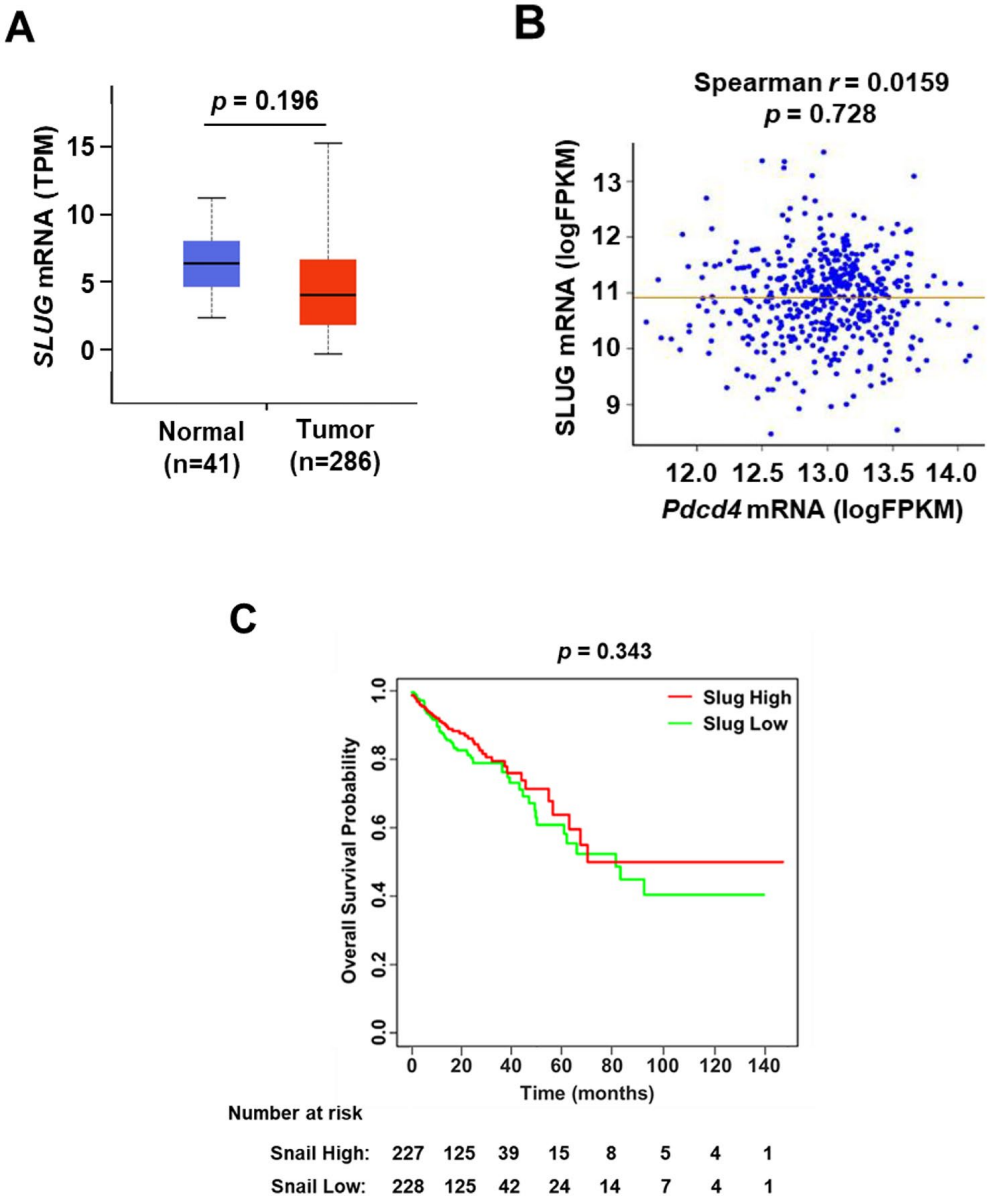
Slug expression in normal and cancer colon tissues. (A) The mRNA level of *SLUG* is similar in normal and cancerous tissues. The normalized value of *SLUG* mRNA was analyzed by two‐sample *t* test (*p* = 0.19). (B) There is no correlation between mRNA level of *SLUG* and *PDCD4*. The normalized values of *SLUG* mRNA and *PDCD4* mRNA were analyzed by Spearman rank correlation test (*r* = 0.0159; *p* = 0.728). (C) Kaplan–Meier overall survival curves and number at risk table for patients with low and high *SLUG* mRNA levels. The Kaplan–Meier curve and log‐rank test were used to compare the overall survival between high and low *SLUG* groups (*p* = 0.343). The estimated 1‐year, 2‐year, 3‐year, 5‐year, and 8‐year survival rates for the *SLUG* mRNA low group are 87.6%, 80.4%, 76.3%, 60.8%, and 40.4%, respectively, compared to 91.1%, 86.1%, 79.5%, 63.8%, and 50.0% for the *SLUG* mRNA high group.

Interestingly, the *SLUG* mRNA levels in colorectal cancer tissues and normal tissues were similar (Figure 2A) and no correlation between *PDCD4* and *SLUG* mRNA level was found [Spearman's correlation coefficient = 0.0159, 95% confidence interval = (−0.081, 0.115), *p* = 0.728] (Figure 2B). Additionally, there was no significant correlation between *SLUG* mRNA levels and patient overall survival (Figure 2C). However, there are no Slug protein data in the existing databases.

### 3.2 Pdcd4 Regulates Slug Expression in Established and Primary Colorectal Cancer Cells

**FIGURE 3:**
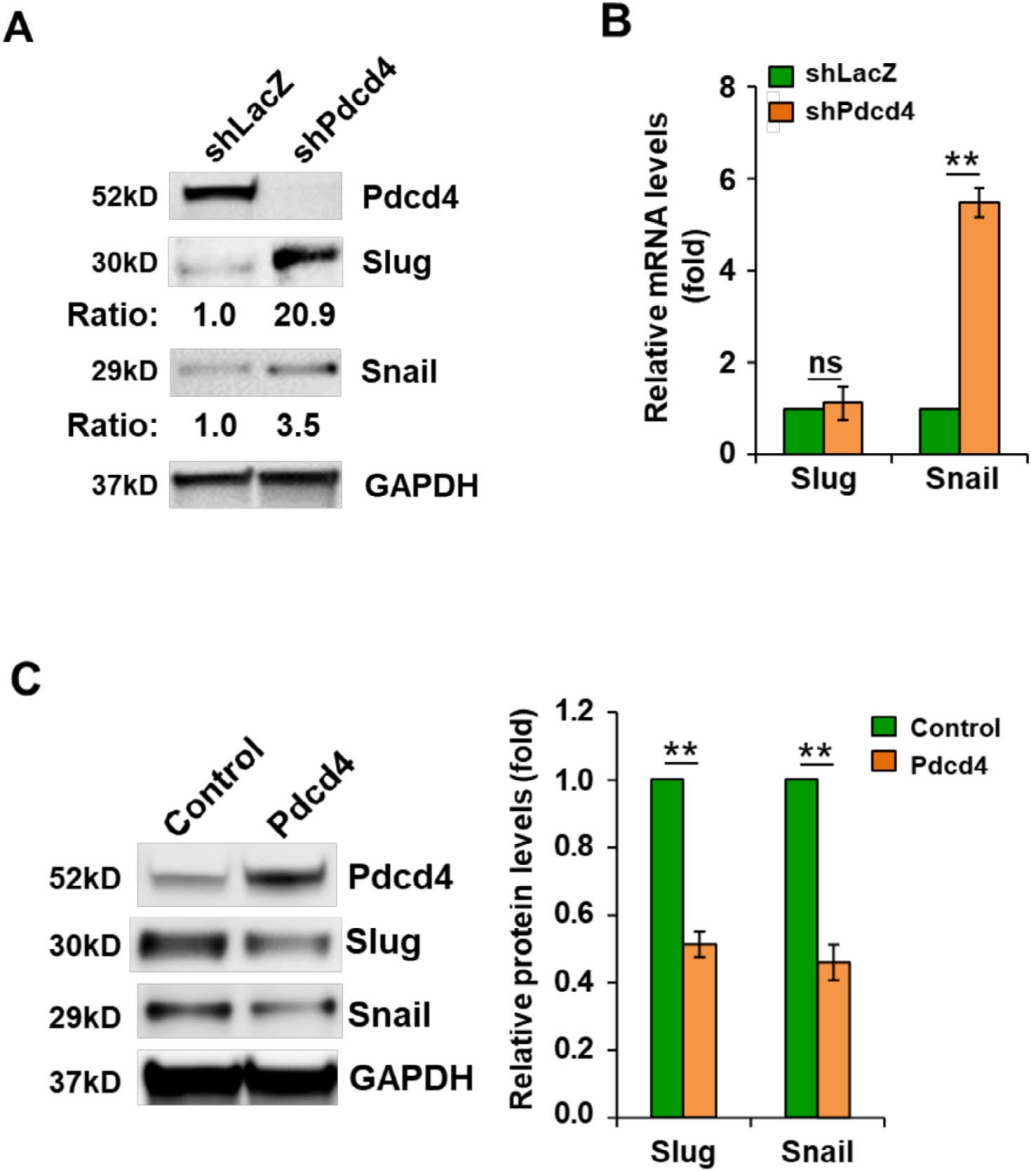
Pdcd4 regulates Slug and Snail expression. (A) Pdcd4 knockdown up‐regulates Slug and Snail protein levels. The band intensity of Slug/GAPDH or Snail/GAPDH in HT29 cells transduced with shLacZ is designated as 1.0. (B) Pdcd4 knockdown does not alter *SLUG* mRNA level. The *SLUG* and *SNAIL* mRNA levels were determined by RT‐qPCR. The level of *SLUG* mRNA or *SNAIL* mRNA in cells transduced with shLacZ is designated as 1.0. Data from three independent experiments were analyzed using a one‐sample *t*‐test (mean ± SD; n.s. = non‐significant; **p < 0.01). (C) Ectopic expression of Pdcd4 decreases Slug and Snail protein levels. Left: Representative immunoblot images. Right: Densitometric quantification of the immunoblot results. The band intensity of Slug/GAPDH or Snail/GAPDH in the control cells is designated as 1.0. Data represent the mean ± SD from three independent experiments and were analyzed using a one‐sample *t*‐test (*p* < 0.01).

Since Pdcd4 knockdown up‐regulates Snail expression [37], it is interesting to examine whether Pdcd4 also regulates Slug expression in colorectal cancer cells. Pdcd4 was knocked down in HT29 cells by transduction of lentiviral particles containing *PDCD4* shRNA. The *PDCD4* shRNA almost completely abolished the Pdcd4 protein level in HT29 cells compared to corresponding *LacZ* shRNA‐transduced (control) cells (Figure 3A). We found that knockdown of Pdcd4 dramatically increased protein levels of Slug and Snail (Figure 3A). Next, we compared the *SNAIL* mRNA levels in the Pdcd4 knockdown and control cells. As shown in Figure 3B, the *SNAIL* mRNA level was significantly increased in Pdcd4 knockdown HT29 cells compared to control HT29 cells (expressing shLacZ) while the *SLUG* mRNAs remained similar in both control and Pdcd4 knockdown HT29 cells. Since HT29 cells express very low levels of Slug protein (Figure 3A) and Pdcd4 is expected to suppress Slug expression, we did not choose HT29 cells for the Pdcd4 overexpression experiments. Instead, we selected the patient‐derived primary colon cancer cells, Pt130, which exhibit high Slug protein levels. As shown in Figure 3C, ectopic expression of resulted in a reduction of Slug and Snail protein levels (Figure 3C), suggesting that Pdcd4 knockdown upregulated Slug expression at the post‐transcription level.

### 3.3 Pdcd4 Knockdown Increases Slug Translation

**FIGURE 4:**
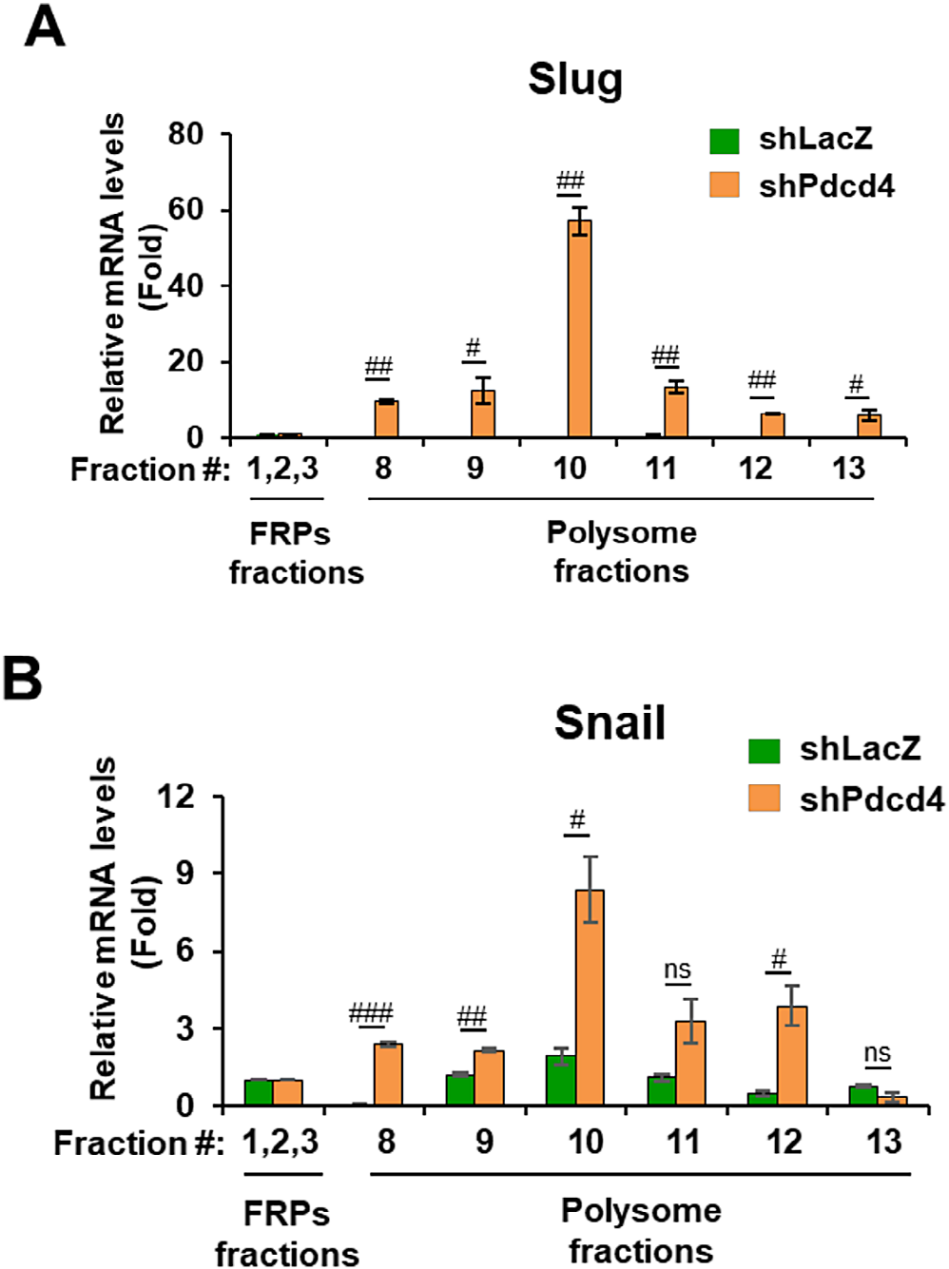
Pdcd4 knockdown enhances translation of Slug and Snail. The mRNA distributions of (A) *SLUG* and (B) *SNAIL* mRNAs in polysome fractions were analyzed using sucrose gradient fractionation. The mRNAs in each fraction were purified and quantified with RT‐qPCR. RT‐qPCR was performed in triplicate to determine the relative levels of *SLUG* or *SNAIL* mRNA by comparing the target mRNA in each polysome fraction to that in the corresponding fraction of pooled free RNAs and proteins (FRP). Data were analyzed using a two‐sample *t*‐test (mean ± SD; ns = not significant; ^#^ *p* < 0.05; ^##^ *p* < 0.01; ^###^ *p* < 0.001).

Pdcd4 has been known to function as an inhibitor of protein translation by binding with translation initiation factor eIF4A and suppressing its activity [29, 31]. Since Pdcd4 knockdown increased Slug protein level and did not alter either *SLUG* mRNA level (Figure 3A,B), it suggests that Pdcd4 knockdown promotes Slug protein translation. To assess it, we performed sucrose gradient fractionation to separate free RNAs and proteins (FRPs), monosomes, and polysomes [29]. The mRNAs in the polysome fractions are the mRNAs that are actively translated. After gradient fractionation, the RNAs in pooled FRPs fractions and each polysome fraction were purified, and the relative amount of *SLUG*, *SNAIL*, or *ATF4* mRNA was quantified by RT‐qPCR. As shown in Figure 4A, the level of *SLUG* mRNA was dramatically increased in polysome fractions (fraction #8 to #13) in Pdcd4 knockdown HT29 cells compared to the corresponding fraction in control cells, indicating that Pdcd4 knockdown in HT29 cells increased the translation of *SLUG* mRNA. We also found that the *SNAIL* mRNA population increased from the polysome fraction #8 to #12 in Pdcd4 knockdown cells compared to the corresponding fraction in control cells (Figure 4B), suggesting that Pdcd4 knockdown also enhances the translation of *SNAIL* mRNA. Our findings (Figures 3 and 4) indicate that Pdcd4 regulates Slug expression at the level of protein translation, while it controls Snail expression at both the transcriptional and translational levels.

### 3.4 The SLUG 5′UTR is an Essential Element for Pdcd4 to Inhibit Slug Translation

**FIGURE 5:**
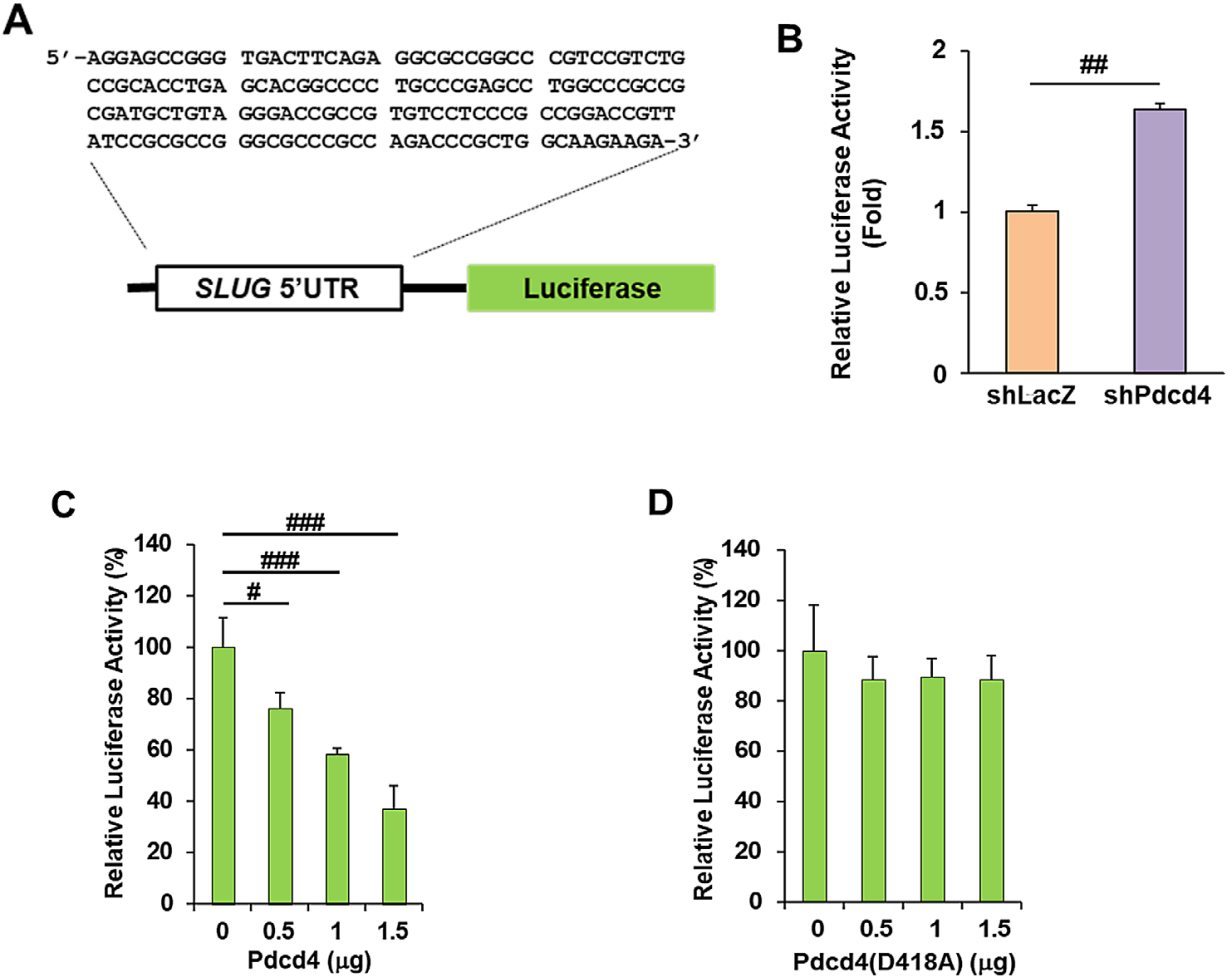
The 5′UTR of *SLUG* mRNA mediates Pdcd4‐inhibited Slug translation. (A) The *SLUG* 5′UTR sequence. (B) Pdcd4 knockdown increases *SLUG*‐5′UTR‐Luc activity. Pdcd4 knockdown (shPdcd4) and control (shLacZ) HT29 cells (5 × 10^4^ per well in 24‐well plate) were co‐transfected with *SLUG*‐5′UTR‐Luc (0.2 μg) and pRL‐SV40 (20 ng). The normalized luciferase activity (firefly luciferase/Renilla luciferase) in control cells is designated as 1. Data were analyzed using a two‐sample t‐test (*n* = 4; mean ± SD; ^##^ *p* < 0.01). (C and D) Pdcd4 (C) but not Pdcd4 (D418A) mutant (D) inhibits *SLUG*‐5′UTR‐Luc activity in a dose dependent manner. RKO cells (5 × 10^4^ per well) were transfected with 0–1.5 μg of pcDNA‐Pdcd4, 0.2 μg of *SLUG*‐5′UTR‐Luc, and 20 ng of pRL‐SV40. The total DNA for each transfection was maintained at 1.7 μg by adding pcDNA 3.1+ vector DNA. The normalized luciferase activity (firefly luciferase/Rinilla luciferase) in cells transfected with 0 μg of *PDCD4* plasmid is designated as 100%. Data were analyzed using a two‐sample *t*‐test (*n* = 4; mean ± SD; ^#^ *p* < 0.05; ^###^ *p* < 0.001).

To examine whether the 5′UTR of *SLUG* mRNA is essential for Pdcd4 to inhibit Slug translation, we cloned the 5′UTR of *SLUG* using 5′RACE, and subsequently ligated it into the pCMV‐Luc plasmid [31] and named it SLUG‐5′UTR‐Luc (Figure 5A). The cloned *SLUG* 5′UTR contains 159 nucleotides with about 75% G + C content, which can form a secondary structure with a free energy of −74.90 kcal/mol (determined by mfold). This SLUG‐5′UTR‐Luc plasmid was then transfected into Pdcd4 knockdown and control cells. The luciferase activity of the SLUG‐5′UTR‐Luc in Pdcd4 knockdown HT29 cells is approximately 1.6‐fold higher than that in control cells (Figure 5B). Next, we transfected the Pdcd4 expressing plasmid along with SLUG‐5′UTR‐Luc into colorectal cancer RKO cells to test whether Pdcd4 inhibits the translation of SLUG‐5′UTR‐Luc. The RKO cells were used for the experiment because they have relatively lower levels of Pdcd4 compared to HT29 and GEO cells [38], thereby reducing interference from endogenous Pdcd4. As shown in Figure 5C, Pdcd4 inhibited SLUG‐5′UTR‐Luc translation in a concentration‐dependent manner. Transfection of 1.5 μg of Pdcd4 expression plasmid inhibited approximately 60% of SLUG‐5′UTR‐Luc activity. In contrast, transfection of Pdcd4 (D418A), a Pdcd4 mutant that is incapable of binding with eIF4A and inhibiting protein translation [29], failed to inhibit SLUG‐5′UTR‐Luc translation in RKO cells (Figure 5D). Since Pdcd4 does not affect luciferase transcription [23] and luciferase enzymatic activity [31], these findings imply that Pdcd4 inhibited SLUG‐5′UTR‐Luc activity through translation suppression. Collectively, our data indicate that the *SLUG* 5′UTR is essential for Pdcd4 to suppress Slug translation.

### 3.5 Suppression of eIF4A Activity Decreases the Level of Slug Protein

**FIGURE 6:**
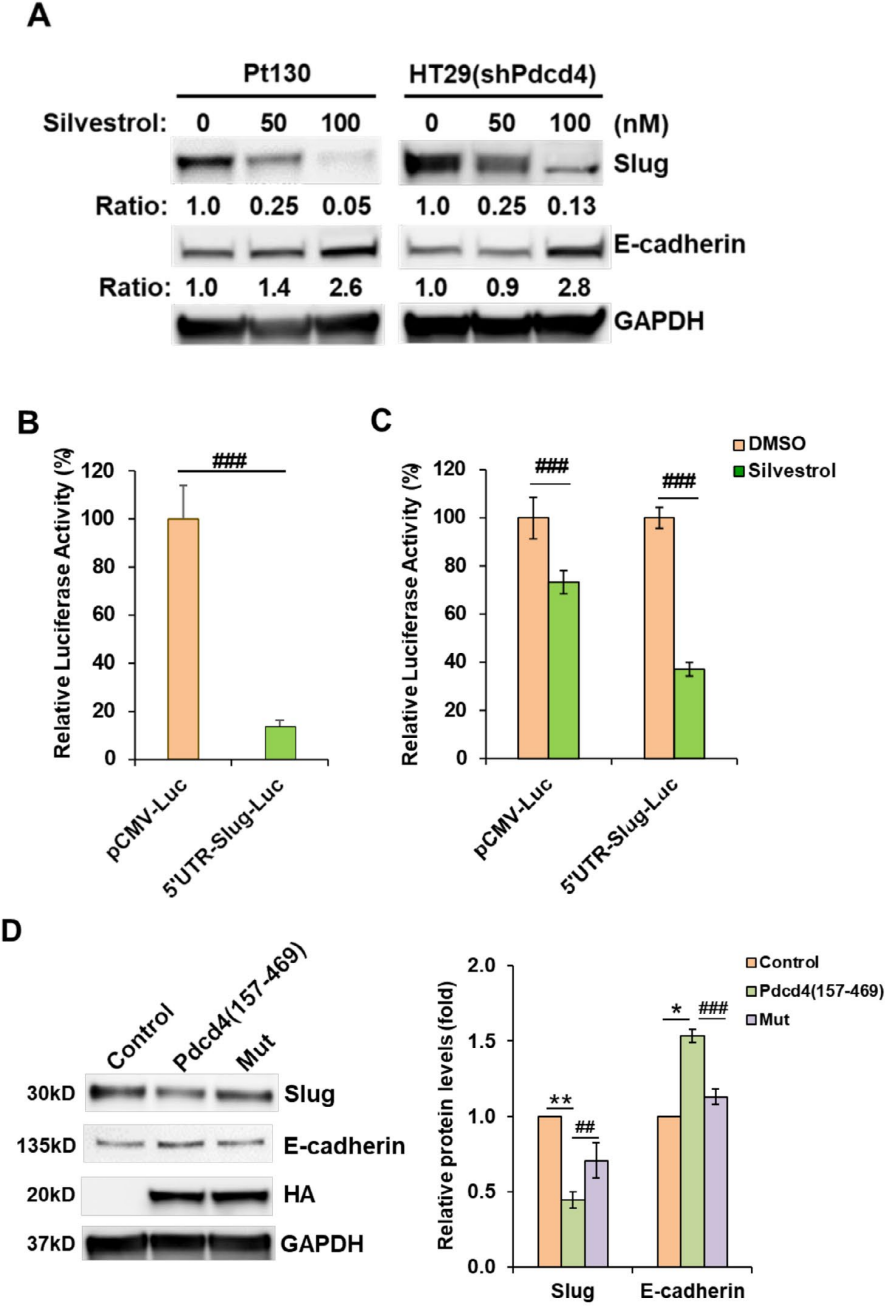
Inhibition of eIF4A decreases Slug translation. (A) Slug protein level inhibited by silvestrol. Pt130 or HT29 (shPdcd4) cells were treated with indicated concentration of silvestrol for 48 h. The ratio of Slug/GAPDH and E‐cadherin/GAPDH in cells treated with DMSO (0 nM silvestrol) is designated as 1.0. (B) Translation of *SLUG*‐5′UTR‐Luc is inefficient. Pt130 cells (8 × 10^4^ per well in 24 well plate) were transfected with 0.2 μg of *SLUG*‐5′UTR‐Luc or pCMV‐Luc along with 20 ng of pRL‐SV40. The normalized luciferase (firefly luciferase/Renilla luciferase) activity in cells transfected with pCMV‐Luc is designated as 100%. Data were analyzed using a two‐sample *t*‐test (*n* = 4; mean ± SD; ^###^ *p* < 0.001). (C) Silvestrol inhibits the *SLUG*‐5′UTR‐Luc translation. Pt130 cells (8 × 10^4^ per well in 24 well plates) were transfected with 0.2 μg of *SLUG*‐5′UTR‐Luc or pCMV‐Luc along with 20 ng of pRL‐SV40. The firefly luciferase/Renilla luciferase activity in cells treated with DMSO (0 nM silvestrol) is designated as 100%. Data were analyzed using a two‐sample *t*‐test (*n* = 4; mean ± SD; ^###^ *p* < 0.001). (D) Pdcd4 (157–469) but not Mut induces E‐cadherin protein expression. Pt130 cells (8 × 10^5^ in 60 mm plate) were transfected with 5.0 μg pcDNA3.1+ (control), Pdcd4 (157–469) expressing plasmid, or Mut expressing plasmid. Seventy‐two hours post‐transfection, cell lysates were collected and subsequently immunoblotted with indicated antibodies. Left: Representative immunoblot images. Right: Densitometric quantification of the immunoblot results. The ratio of Slug/GAPDH and E‐cadherin/GAPDH in control cells is designated as 1.0. Data from four independent experiments comparing control and Pdcd4 (157–469) were analyzed using a one‐sample *t*‐test (mean ± SD; *p < 0.05; **p < 0.01). Comparisons between Pdcd4 (157–469) and Mut were analyzed using a two‐sample *t*‐test (mean ± SD; ^##^ *p* < 0.01; ^###^ *p* < 0.001). Mut: Pdcd4 (157–469) (D253A, D418A).

To explore whether the mechanism of Pdcd4 suppressing Slug translation is through inhibition of eIF4A, the cells were treated with an eIF4A inhibitor, silvestrol. As shown in Figure 6A, Pt130 and Pdcd4 knockdown HT29 cells treated with silvestrol decreased the Slug protein level in a concentration‐dependent manner. Cells treated with 100 nM of silvestrol reduced the Slug expression approximately 20‐ and 7.7‐fold in Pt130 and Pdcd4 knockdown HT29 cells, respectively, compared to the untreated cells. As the consequence of the inhibited Slug expression, the expression of E‐cadherin, the Slug‐inhibited downstream target, is elevated approximately 2.8‐fold and 2.6‐fold in Pt130 and Pdcd4 knockdown HT29 cells, respectively, in cells treated with 100 nM of silvestrol. To further confirm that eIF4A regulates Slug expression through *SLUG* 5′UTR, the Pt130 cells were transfected with SLUG‐5′UTR‐Luc or non‐structured luciferase reporter (pCMV‐Luc) and subsequently treated with 100 nM of silvestrol. As shown in Figure 6B, translation of the structured SLUG‐5′UTR‐Luc is relatively inefficient, exhibiting only 15% of the luciferase activity compared to the non‐structured pCMV‐Luc construct. Silvestrol at 100 nM inhibited approximately 65% of the translation of the structured SLUG‐5′UTR‐Luc, while it suppressed only about 25% of the translation of the non‐structured pCMV‐Luc (Figure 6C). These results suggested that the efficiency of the translation of *SLUG* mRNA depends on eIF4A activity.

Crystal structure analyses show that Pdcd4 (157–469) fragment, containing two MA‐3 domains within 157 to 469 amino acid residues, binds to eIF4A and sufficiently inhibits protein translation [28, 39]. Pdcd4 has been reported to bind with RNA, phosphorylated by Akt, and methylated by protein arginine methyltransferase 5 in the region of 1 to 156 amino acid residues [40]. To avoid the interference of elements other than MA3 domains, we used Pdcd4 (157–469) and the eIF4A binding defective variant Pdcd4 (157–469) (D253A, D418A) to examine the impact of Pdcd4 on Slug and E‐cadherin expressions. The Pdcd4 (157–469) and Pdcd4 (157–469) (D253A, D418A) expression plasmids were transfected into Pt130 cells, and subsequently, the Slug and E‐cadherin protein levels in the transfected cells were assessed with immunoblotting. Over‐expression of Pdcd4 (157–469) led to about a 55% reduction in Slug protein level and an approximately 1.5‐fold increase in E‐cadherin protein level compared to control cells transfected with an empty vector (Figure 6D). However, the cells expressing Pdcd4 (157–469) (D253A, D418A) mutant only reduced about 15% of Slug protein abundance. The E‐cadherin protein levels were similar between the Pdcd4 (157–469) (D253A, D418A) expressing cells and control cells. Together, these results indicated that Pdcd4 suppresses Slug translation through inhibition of eIF4A activity.

### 3.6 Up‐Regulation of Slug by Pdcd4 Knockdown Contributes to Invasion Promotion

**FIGURE 7:**
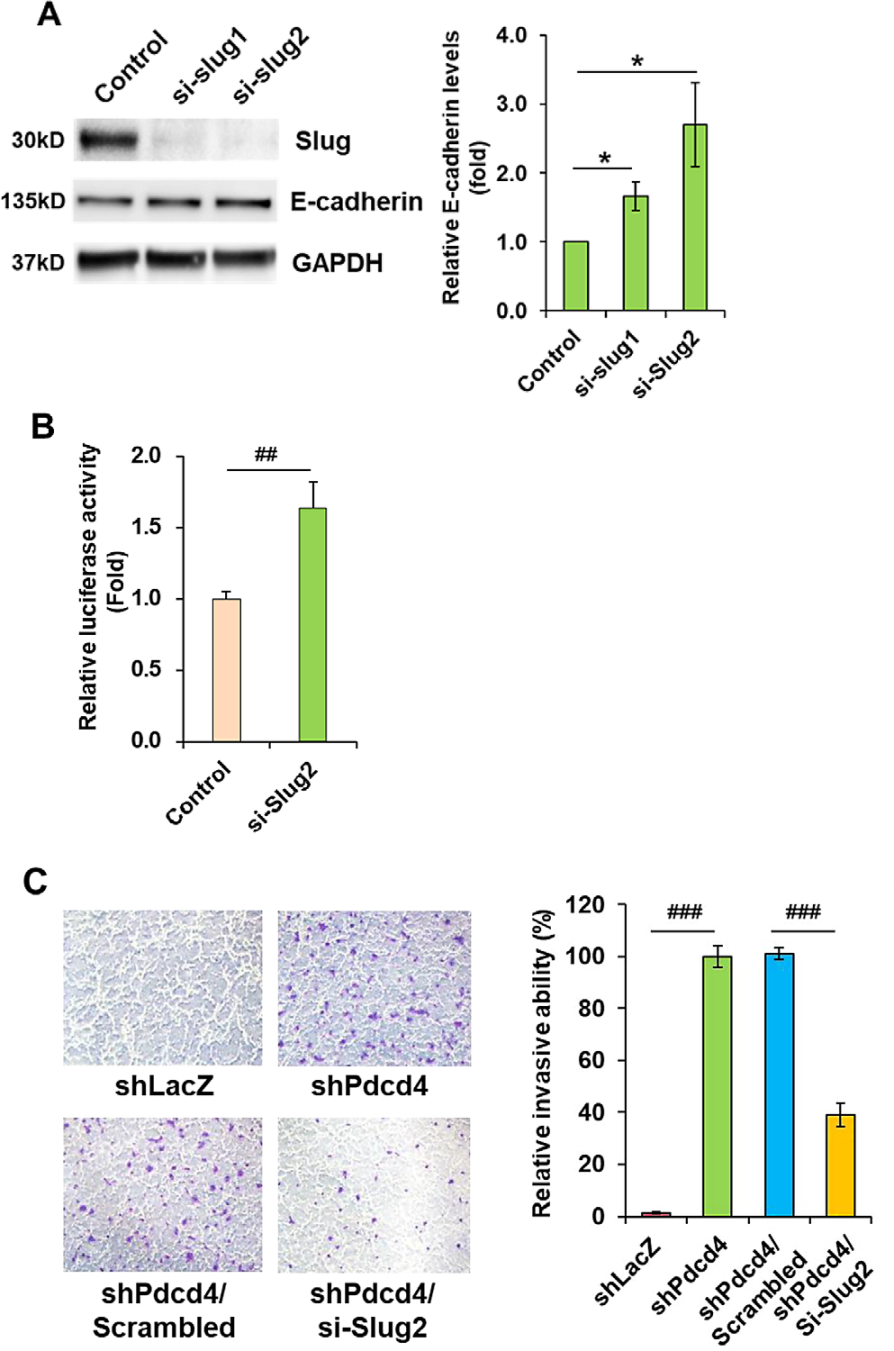
Knockdown of Slug reverses the invasive capability induced by Pdcd4 knockdown. (A) Slug knockdown increases E‐cadherin expression in HT29 knockdown cells. The Slug expression was knocked down by two commercial *SULG* siRNAs in Pdcd4 knockdown HT29 cells and the cell lysates were subjected to immunoblot analysis. The ratio of E‐cadherin/GAPDH in scrambled siRNA transfected cells (control) is designated as 1.0. Data from three independent experiments were analyzed using a one‐sample *t*‐test (mean ± SD; *p < 0.05). (B) Slug knockdown restores the E‐cadherin promoter activity in Pdcd4 knockdown HT29 cells. The cells were transfected with 0.2 μg of E6 E‐cadherin promoter plasmid, 0.3 μg of pcDNA3.1, and 20 ng of pRL‐SV40. The luciferase activity was normalized against Renilla activity. The activity of scrambled siRNA transfected cells (control) is designated as 1.0. Data were analyzed using a two‐sample *t*‐test (*n* = 5; mean ± SD; ^##^ *p* < 0.01). (C) Slug knockdown in Pdcd4 knockdown HT29 (shPdcd4) cells relieves invasive induction by Pdcd4 knockdown. The cells were serum starved for 24 h and subsequently assayed for invasive capability. Left, representative images of Matrigel invasion. Right, quantification of invasion. The number of invasive cells in Pdcd4 knockdown HT29 (shPdcd4) cells is designated as 100%. Data were analyzed using a two‐sample *t*‐test (*n* = 3; mean ± SD; ^###^ *p* < 0.001).

Next, we asked whether the elevation of Slug by Pdcd4 knockdown is functionally significant in promoting cell invasion. If Pdcd4 knockdown increases Slug translation to promote cell invasion, then directly down‐regulating Slug expression in the Pdcd4 knockdown cells should reduce the invasive ability of the cells. To test this, we knocked down Slug expression using two *SLUG* siRNAs in Pdcd4 knockdown HT29 cells and examined the effect on E‐cadherin expression. The *SLUG* siRNAs successfully reduced the Slug protein levels by more than 90%, and concomitantly, the E‐cadherin protein levels increased about 1.7‐ to 2.7‐fold (Figure 7A). In consistency with the immunoblot results, the knockdown of Slug in Pdcd4 knockdown cells increased approximately 1.7‐fold in E‐cadherin promoter activity (Figure 7B). As shown in Figure 7C, the invasive capability dramatically increased in Pdcd4 knockdown HT29 cells as compared to control cells, which was partially inhibited as Slug expression was down‐regulated. The invasive capability of Pdcd4 knockdown cells transfected with *SLUG* siRNA was reduced to approximately 45% compared to Pdcd4 knockdown cells transfected with scrambled siRNA, indicating that Slug up‐regulation caused by Pdcd4 knockdown contributes to invasion promotion in colorectal cancer cells.

## 4 Discussion

In the present study, we establish that Pdcd4 knockdown promotes *SLUG* mRNA translation (Figure 4) through eIF4A mediated by *SLUG* 5′ UTR (Figures 5 and 6). We also show that the up‐regulation of Slug by Pdcd4 knockdown is functionally significant in terms of down‐regulating E‐cadherin expression and promoting cell invasion in colorectal cancer cells (Figure 7). Thus, our results indicate that translational up‐regulation of Slug expression by Pdcd4 knockdown contributes to invasion promotion in colorectal cancer cells.

Pdcd4 has been demonstrated to translationally regulate the expression of Sin1 and p70S6K1 by suppression of eIF4A activity [23, 24]. Here, we further identify Slug as a direct translational target of Pdcd4 via eIF4A. Pdcd4 is the binding partner of eIF4A and inhibits eIF4A's helicase activity. By using eIF4A variants that are defective in RNA helicase, Svitkin et al. showed that translations of structured mRNAs by eIF4A variants with helicase defects are not as efficient as those of less structured mRNAs [41]. Thus, the translation of structured mRNAs is highly dependent on eIF4A's helicase activity and should be susceptible to inhibition by Pdcd4. This notion is further supported by our findings that the translation of SLUG‐5′UTR‐Luc is less effective than that of non‐structured pCMV‐Luc and is sensitive to eIF4A inhibition (Figure 6). We also found that the free energy of the 5′UTR mRNA is critical for Pdcd4‐mediated translational inhibition. Previously, we demonstrated that Pdcd4 efficiently inhibits translation of luciferase mRNA with an artificial stem‐loop structure at the 5′UTR with a free energy of −44.8 kcal/mol [31], suggesting that Pdcd4 preferentially inhibits translation of 5′UTR structured mRNA with a free energy greater than −44.8 kcal/mol. This concept is further confirmed by the findings that Pdcd4 regulates the translation of Sin1 [23], p70S6K1 [24], and Slug (Figure 5) in which the free energies of the 5′UTRs are −145 kcal/mol, −82.30 kcal/mol, and −74.90 kcal/mol, respectively. Thus, knockdown of Pdcd4 increases Slug translation but has no impact on its transcription (Figures 3 and 4). However, the minimal free energy of the 5′UTR mRNA for Pdcd4 translational inhibition is unknown, requiring identification and analysis of additional Pdcd4 targets. In addition to eIF4A‐dependent translation inhibition, Pdcd4 can also inhibit translation through an eIF4A‐independent mechanism [22]. For example, Pdcd4 may directly bind with *XIAP* mRNA and block the translational initiation complex formation [26]. However, it is unclear whether Pdcd4 binds to mRNA through a specific secondary structure or a nucleotide motif, requiring further examination.

The findings that Slug knockdown partially reverses the effects of Pdcd4 knockdown in elevating E‐cadherin expression and repressing invasion suggest that Slug contributes to Pdcd4 knockdown‐mediated tumor invasion (Figure 7). How does Pdcd4 knockdown up‐regulate Slug protein abundance to promote invasion? Up‐regulation of Slug by Pdcd4 knockdown led to down‐regulation of E‐cadherin and subsequently led to β‐catenin translocation into nuclei to stimulate transcription of c‐Myc and urokinase‐type plasminogen activator receptor (u‐PAR) [37]. The u‐PAR is a membrane‐bound glycoprotein. Upon binding to its ligand, urokinase plasminogen activator, u‐PAR triggers a proteolytic cascade leading to the degradation of the extracellular matrix. This degradation allows tumor cells to penetrate the basal membrane during invasion [42]. The c‐Myc is an oncogene and transcription factor, which stimulates the expression of mitogen‐activated protein kinase kinase kinase kinase 1 to activate the JNK/AP‐1 axis for promoting tumor invasion [43].

Pdcd4 knockdown increases Slug protein level but not *SLUG* mRNA level, and enhances the *SLUG* mRNA population in polysome fractions, indicating that Pdcd4 regulates Slug expression at the translation level (Figures 3 and 4). In contrast, Pdcd4 knockdown dramatically increases *SNAIL* mRNA and protein (Figure 3). These findings suggest that Pdcd4 regulates Snail and Slug expression through different mechanisms. One possible mechanism for the regulation of Snail transcription by Pdcd4 is through the mammalian target of rapamycin complex 2 (mTORC2)‐Akt‐NFκB axis. Pdcd4 knockdown has been shown to repress Sin1 translation and thereby inactivate mTORC2 [23]. Phosphorylation of Akt at Ser473 by mTORC2 enhances Akt kinase activity [44], which in turn increases the phosphorylation of NF‐κB inhibitor, IκB, resulting in the degradation of IκB and activation of NF‐kB to increase Snail transcription [23, 35, 45]. In addition, Evdokimova et al. reported that *SNAIL* mRNA forms a secondary structure at 5′UTR, which facilitates the binding with Y‐box binding protein (YB‐1) to regulate the translation of *SNAIL* mRNA [46]. Our findings that Pdcd4 knockdown increases the populations of *SNAIL* mRNA in polysome fractions suggested Snail translation may also be regulated by Pdcd4. It is unknown whether Pdcd4 inhibits Snail translation through the facilitation of YB‐1 binding to *SNAIL* mRNA or through inhibition of eIF4A activity, which needs further investigation.

How does Pdcd4 suppress colon tumorigenesis through inhibition of eIF4A activity? Using genome‐wide ribosome profiling, eIF4A was found to regulate the translation of a set of eIF4A‐dependent mRNAs [47]. The eIF4A‐dependent mRNAs frequently encode proteins for cell cycle progression, cell survival, and cell migration [48]. Therefore, suppression of eIF4A activity is likely to inhibit tumorigenesis. Our findings that Pdcd4 but not the eIF4A binding defective variant suppresses Slug expression and cell invasion (Figure 7) provide evidence for inhibiting eIF4A to suppress invasion. It has been reported that down‐regulation of Slug results in suppression of proliferation in cultured cells and tumor growth in mice [49]. Therefore, inhibition of Slug translation by Pdcd4 results in suppression of colorectal cancer cell proliferation and invasion. Besides, we discovered that Pdcd4 inhibits translation of Sin1, a critical component of the mTORC2, through suppression of eIF4A activity. mTORC2 comprises mTOR, Rictor, Sin1, Protor‐1, and Deptor, which phosphorylates Akt, SGK, and PKCα to govern cell survival, motility, and invasion [50, 51]. The expression of mTORC2 components such as Rictor and mTOR is frequently up‐regulated in colorectal cancer patients [52] and knockdown of mTORC2 components results in suppression of cell proliferation and invasion [53]. Hence, inhibition of eIF4A activity by Pdcd4 is expected to repress the translation of a set of mRNAs to attenuate tumorigenesis.

This results of this study suggests two potential therapeutic approaches for metastatic colorectal cancer: (1) restoring PDCD4 expression using nanoparticle‐delivered PDCD4 cDNA/mRNA or natural compounds such as resveratrol [54], and (2) suppressing eIF4A activity with agents like silvestrol [55]. For example, PDCD4 restoration may be prioritized in tumors with low PDCD4 expression, while eIF4A inhibitors could be more effective in patients with high eIF4A levels. Although nanoparticle‐mediated delivery of cDNA or mRNA has been evaluated in clinical trials, the specific efficacy of PDCD4 cDNA/mRNA delivery via nanoparticles remains untested and warrants further investigation.

## 5 Conclusions

This study demonstrated that the Pdcd4 regulates Slug protein translation to control E‐cadherin expression and invasion in colorectal cancer cells. In addition, inhibition of eIF4A activity by silvestrol significantly reduced Slug translation, providing a promising therapeutic approach for colorectal cancer.

## Author Contributions

H.‐S.Y. and Q.W. designed the experiments and wrote the manuscript. Q.W., S.H., W.H., Y.W., L.C., and J.Z. performed the experiments. Q.W., M.L., Y.Z., J.Z., and H.‐S.Y. interpreted data and edited the manuscript.

## Consent

Informed consent was obtained from all subjects involved in the study.

## Conflicts of Interest

The authors declare no conflicts of interest.

## Data Availability

The data that support the findings of this study are available from the corresponding author upon request.
